# Forensic integrity of blood alcohol sampling in the emergency department, Part II: Establishing quality goals of turnaround time with Monte Carlo simulation

**DOI:** 10.11613/BM.2026.030704

**Published:** 2026-08-10

**Authors:** Cristiano Ialongo

**Affiliations:** Department of Clinical Pathology, University Hospital Policlinico Umberto I, Rome, Italy

**Keywords:** blood alcohol concentration, Monte Carlo method, healthcare quality indicators, forensic toxicology, computer simulation

## Abstract

**Introduction:**

In the emergency department (ED), the turnaround time (TAT) for forensic blood alcohol sampling is a critical determinant of legal admissibility. Due to the pharmacokinetics of ethanol, delays in sampling lead to metabolic decay, potentially resulting in pharmacokinetic false negatives (FN_pk_). This study aims to establish objective, evidence-based quality goals for intra-ED TAT using Monte Carlo simulation to preserve forensic reliability.

**Materials and methods:**

Data were collected from 121 forensic cases at a major metropolitan university hospital in 2025. A Monte Carlo simulation (N = 50,000 iterations) was performed to model the probability of FN_pk_ based on ethanol elimination rate (β), analytical imprecision (CV), and extra-ED *versus* intra-ED TAT components. Probability density functions (PDFs) were fitted using Weibull and Gamma distributions to characterize observed clinical and logistical data.

**Results:**

The simulation revealed a baseline P(FN_pk_) of 7.0% for the intra-ED phase, increasing to 7.6% when including extra-ED transport. Quality goals were derived for three tiers: optimal (TAT_95%_) at 134 minutes, acceptable (TAT_80%_) at 214 minutes, and minimum (TAT_60%_) at 273 minutes. Regression analysis showed that for every 1% increase in assay CV, the TAT quality goal erodes by 2.7 to 3.3 minutes. The logistic process was found efficient, with an Index of Logistic Reliability (I_LR_) of 86.4%.

**Conclusions:**

Monte Carlo simulation effectively translates pharmacokinetic variables into actionable forensic quality goals. Current ED performance requires process improvement, particularly in the request-transmission phase, to minimize FN_pk_ and ensure forensic equity.

## Graphical abstract

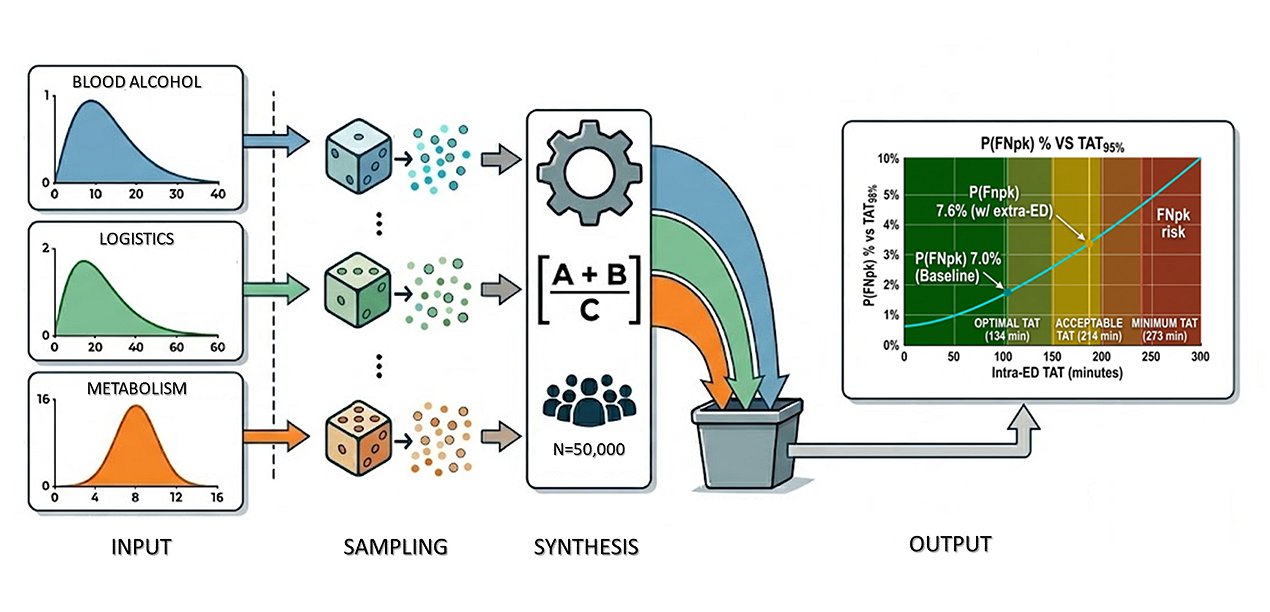

## Highlights

• Monte Carlo simulation reveals a 7.0% risk of false negatives in the emergency department (ED)

• Optimal quality goal for intra-ED sampling is set at 134 minutes (95% reliability)

• Acceptable and minimum performance goals are 214 and 273 minutes, respectively

• Every 1% increase in assay imprecision erodes the forensic window by 3 minutes

• The Index of Logistic Reliability (ILR) validates transport forensic performance

## Introduction

In the high-pressure environment of the emergency department (ED), timeliness is synonymous with life-saving care. However, in cases involving suspected drink-driving or substance-facilitated crimes, timeliness is critical during the intra-ED preanalytical phase for blood samples collected for forensic purposes. Indeed, together with the integrity of the chain of custody (CoC), the rapidity in blood sampling preserves the significance of the toxicological evidence and thus is inextricably linked to the “legal admissibility” in judicial proceedings (*1*).

Central to monitoring this efficiency is the turnaround time (TAT). Traditionally defined as the interval from the initial diagnostic request to the final validation of results, TAT serves as a primary key performance indicator (KPI) for laboratory and emergency services (*2*). In the forensic context, the narrow window for specimen collection dictated by the pharmacokinetics of ethanol and other drugs entails shortest TAT for avoiding altering evidence due to delay.

To drive process improvement through KPIs, organizations must establish clear quality goals and performance specifications (*3*). Within the ED, however, there is a notable absence of objective benchmarks specifically tailored for forensic sampling. To bridge this gap, an evidence-based model is required to define the relationship between pharmacokinetic variables and statistical metrics of timeliness, such as the median and the 95th percentile of TAT.

This study is a second part of a two-part investigation and directly builds upon the empirical evidence established in Part I (*4*). In the preceding epidemiological paper, a retrospective analysis of 115 forensic cases demonstrated that the median intra-ED preanalytical turnaround time was 139 minutes. Crucially, this workflow velocity remained exceptionally stable and unaffected by operational stressors such as emergency department overcrowding, shifts, or weekends, suggesting a fixed or potentially “degraded” operational baseline. When factoring in transit delays from the scene of the accident, the overall median preanalytical lag reached 204 minutes.

To translate these descriptive findings into actionable clinical and legal quality goals, the present study utilizes the exact data parameters and structural definitions derived from the Part I cohort. Specifically, the empirical probability density functions (PDFs) describing serum alcohol concentrations at admission (SAC_ED_), intra-ED logistical intervals (TAT_intra_), and extra-ED transit timelines (TAT_extra_) are extracted directly from Part I’s dataset to ground our simulation in real-world clinical behavior. By merging these real workflows with established kinetic behaviors of ethanol metabolic decay and assay imprecision, this work aims to execute a Monte Carlo simulation. The objective is to define precise math-driven quality goals for timeliness, establishing clear thresholds where the probability of a pharmacokinetic decay of the sample is minimized to safeguard the legal admissibility of forensic evidence.

## Materials and methods

### Materials

The data used for this simulation were collected from January 1st to December 31st, 2025, at the Azienda Ospedaliero-Universitaria Policlinico Umberto I of Rome (ethical committee approval on laboratory data retrospective analysis nr.0518/2024). They pertain to drivers involved in road accidents for whom forensic toxicological testing was requested, in compliance with the provisions of the Italian Highway Code (*Codice della Strada*). All the specimens were collected and processed in the CoC. Data were presented and discussed in Part I of this series (*4*).

The blood alcohol values used in this study refer to serum alcohol concentration (SAC), in accordance with forensic sample screening procedures performed using the enzymatic method with the alcohol dehydrogenase (ADH) reagent, as described in Part I (*4*). To this regard, according to the Italian Highway Code, as sample testing SAC < 0.5 g/L is considered legally “negative”, and undergone no further confirmatory analysis by headspace-gas chromatography (HS-GC), except for the novice drivers (driving license < 3 years) for whom it is applied the zero-tolerance.

### Methods

#### General principle

The objective of a Monte Carlo simulation is to characterize the behavior of an unknown output variable Y based on N uncertain input variables X_n_. Given a general functional model:



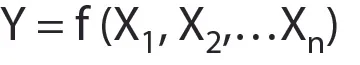



the simulation generates synthetic data to approximate the probability density function (PDF) of Y. The process begins by defining the deterministic relationship between variables and assigning a specific PDF to each input X. During each of the N iterations, a stochastic value is sampled for each input form the PDF and propagated through the model to calculate a point value for Y. Collectively, these N iterations constitute the empirical realization of Y. If N is sufficiently large (*e.g.*, N >10,000), the resulting empirical distribution converges toward the true PDF of the output.

#### Simulation model

If SAC_0_ and SAC_1_ are two concentration values measured at a time interval t from each other, then for SAC_1_ > 0.21 g/L and a known elimination rate β, the relationship can be expressed as:



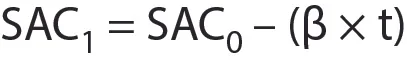



If SAC_0_ = SAC_crit_ is the concentration value at the time of the event of interest (*e.g.*, the car accident) and SAC_1_ = SAC_ED_ is the concentration value measured at the ED, then t = TAT, and we can write:



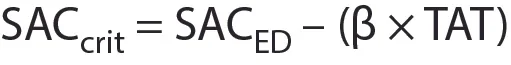



Since SAC_ED_ is subject to measurement error ε due to analytical imprecision (represented by the coefficient of variation, CV) of the assay, the term must be adjusted for each simulation iteration “i” as follows:



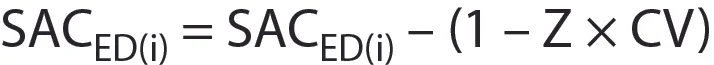



where SAC_ED(i)_ is the i-th simulated value of SAC_ED_, Z represents the standard normal deviate, *i.e.*, a Gaussian distribution with a mean of 0 and unit variance (corresponding to a given level of probability). See Appendix A for further explanation.

Similarly, TAT can be adjusted for the i-th simulation by decomposing into an extra- (TAT_extra_) and an intra-ED (TAT_intra_) component, namely:



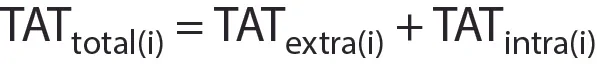



Hence, it follows that:



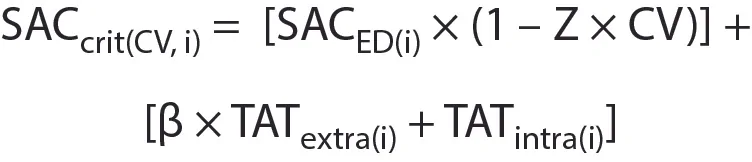



#### Probability of false negatives

Given L as the legal limit for alcohol intoxication (LLI), the i-th subject with a SAC greater than L at the moment of blood sampling in the ED is defined as:



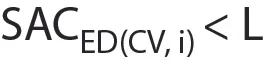



However, an individual with SAC greater than L at the time of the event is defined by Eq.3 as:



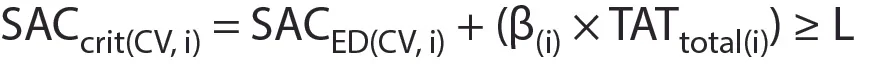



Therefore, an individual rendered “negative” due to an extended TAT satisfies both Eq. 4 and Eq. 5, by definition. Thus, for a pharmacokinetic false negative (FN_pk_), we can write:



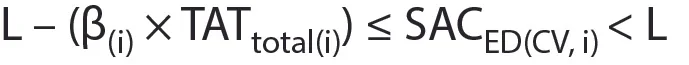



The conditional probability of an FN_pk_ can then be calculated as the ratio of subjects who were formerly positive but are currently negative to the total number of subjects who were formerly positive:



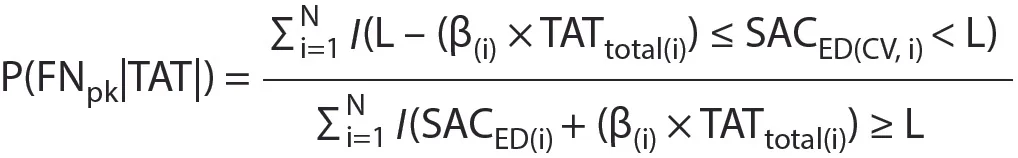



The function *I*(…) represents the indicator function, which returns 1 if the condition within the brackets is met and 0 otherwise. Obviously, the term TAT can be treated according to Eq. 3c.

To provide an objective assessment of FN_pk_, a predefined acceptability threshold (α) can be established:



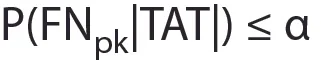



#### Quality goal of turnaround time

If C_0_ = C_road_ is a known or assumed population characteristic (*e.g.*, blood alcohol by roadside breath testing) and C_1_ = L, the quality goal for TAT can be defined based on the “erosion” (metabolic degradation) of ethanol measured in the ED sample:



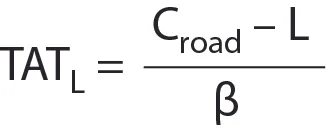



To account for the analytical imprecision of the measurement method in the ED, Eq. 9a can be modified by incorporating the CV, as seen in Eq. 3b:



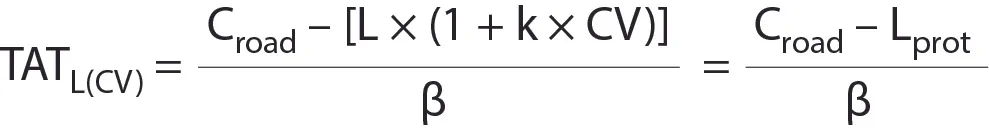



In this case, the random Z is replaced by a constant k, chosen to represent a specific coverage probability for L to provide a “protected” LLI (L_prot_). This serves as a safety threshold to ensure that - net of instrumental error - the subject’s SAC (or BAC) is truly above the legal limit (see Appendix B for further details).

Since Eq. 9b represents the total TAT from the time of the event to the completion of sample collection, it includes both extra-ED and intra-ED time. Therefore, if the PDF of the extra-ED TAT is known, it follows that:



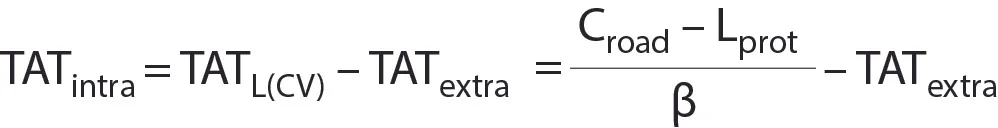



Based on Eq. 9c, quality specifications can be derived from the percentiles of the generated TAT distribution. Consequently, the p-th percentile represents the maximum allowable time to prevent C_road_ from eroding below the legal threshold for 100 x (1-p)% of subjects:



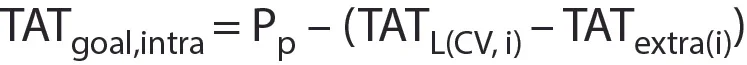



Alternatively, to represent the worst-case scenario (*i.e.*, considering subjects with the highest metabolic degradation rate), the 95th percentile of β can be used in place of the stochastic value:



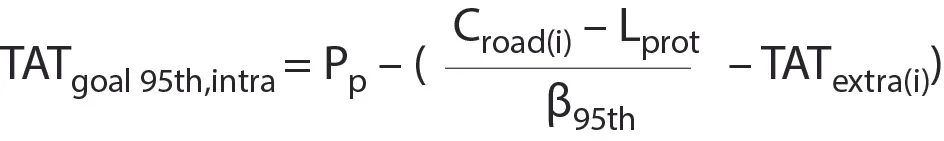



Consequently, quality goals can be directly derived from the 5th (optimal or TAT_95%_), 20th (acceptable or TAT_80%_), and 40th (minimum or TAT_60%_) percentiles of the TAT_goal_. Specifically, the p-th percentile represents the TAT value ensuring that 100 x (1-p)% of the C_road_ distribution remains above the limit L at the time of ED sampling. This value also corresponds to a threshold C_road_ value, which represents the minimum concentration guaranteed to avoid the risk of FN_pk_.

#### Benchmark of extra-ED turnaround time

If TAT_extra_ and C_road_ are known, based on Eq. 9a, an Index of Logistic Reliability (I_LR_) can be derived relative to the average logistic quality:



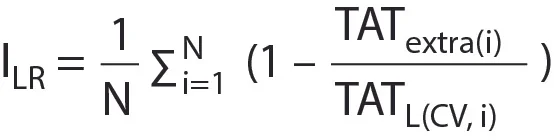



As shown, I_LR_ > 0.5 indicates an efficient logistic service, as at least 50% of TAT_L(CV)_ is available to the ED to complete blood sampling. Conversely, I_LR_ > 0.5 indicates logistic inefficiency, as transport leaves less than 50% of TAT_L(CV)_ for blood sampling. Notably, if TAT_extra_ is so delayed that it exceeds TAT_L(CV)_, then I_LR_ ≤ 0. In such cases, a logistic failure occurs because the patient arrives at the ED when the alcohol level in blood is already below L.

Finally, given Eq. 11, the probability of logistic success (P_LS_) can be calculated as follows:



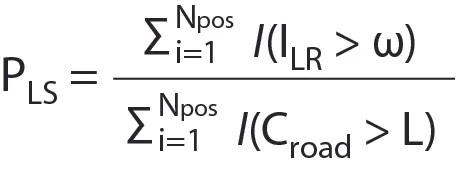



This index returns the probability that the TAT_extra_ is short enough to leave at least 100 x ω% of the TAT_L(CV)_ to the ED personnel to complete the blood sampling before the concentration falls below L_(CV)_.

#### Probability density function estimation

To model the SAC_ED_, TAT_intra_, TAT_extra_, we utilized 3-parameter Lognormal, Weibull, and Gamma distributions. These specific PDFs were selected to accommodate the positive lower bounds and long right tails characteristic of the observed data. The optimal PDF fit was determined by minimizing the sum of squared differences (SSQ) between the empirical and calculated 5^th^, 10^th^, 25^th^, 50^th^, 75^th^, 90^th^ percentiles, as defined by:



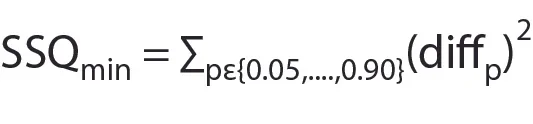



To ensure the distributions respected physical and physiological constraints, a minimum threshold θ) was assigned during parameter optimization. For PDF of SAC_ED_, θ ≥ 0.21 g/L to represent the transition between zero- and first-order kinetics, accounting for a 15% inherent overestimation in SAC measurement (1.15 x 0.18 g/L ≈ 0.21 g/L). Based on empirical evidence, the thresholds for the PDF of TAT_intra_ and the PDF of TAT_extra_ were set at θ ≥ 60 and θ ≥ 30 minutes, respectively.

Conversely, the PDF of β was directly modeled using a Normal distribution based on established literature, with a mean (μ) of 0.15 g/L and a standard deviation (SD) of 0.03 g/L (*5*). The PDF of C_road_ distribution was modeled using a truncated Normal distribution, consistent with established literature on roadside alcohol control (*6*, *7*). The parameters were adjusted to yield a SAC with μ = 1.6 g/L (equivalent to 1.36 g/L BAC), with a SD = 0.30 g/L and θ = 0.30 g/L (0.26 g/L BAC).

### Statistical analysis

Fitting of PDFs and Monte Carlo simulations were conducted using Microsoft Excel 2013 (Microsoft Corporation, Redmond, USA), using Visual Basic macros (which can be provided as add-ins on request). All simulations were performed with N = 50,000 iterations, for CV = 5% and L = 0.5g/L.

The temporal erosion rate of the TAT quality goals relative to the assay CV was determined using ordinary least-squares (OLS) linear regression. To generalize this relationship across different tiers, each quality goal dataset (TAT_95%_, TAT_80%_, and TAT_60%_) was normalized *via* zero-centering. Specifically, the calculated intercept was subtracted from each observed value to force the regression through the origin (*i.e.*, setting the intercept c = 0).

## Results

**Figure 1:**
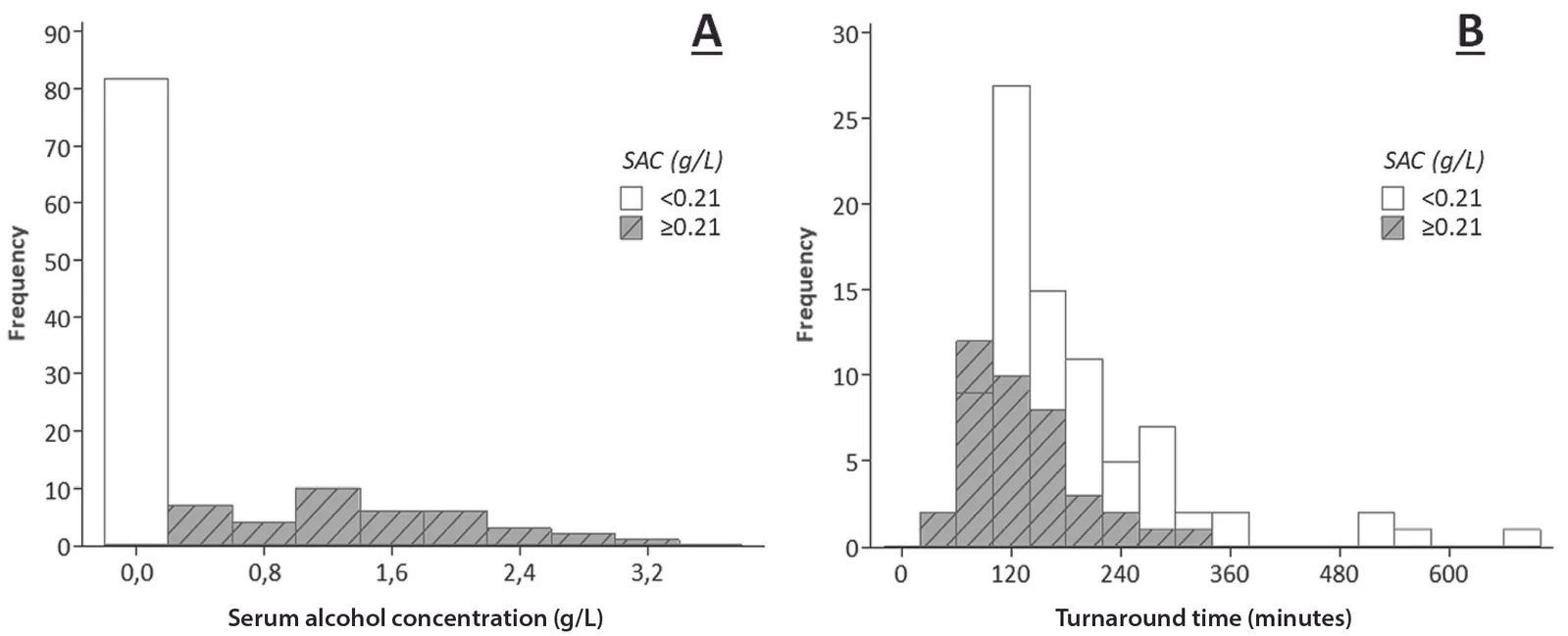
Distribution of serum alcohol concentration (SAC) and turnaround time (TAT) of data selected for Monte Carlo simulations (shaded bars) and excluded data (white bars), based on the eligibility threshold of SAC ≥ 0.21 g/L; panel “A” shows SAC values across the dataset and panel “B” of TAT values, measured in minutes, categorized by the same SAC threshold.

For the 2025 calendar year, the total caseload consisted of N = 121 forensic toxicological cases performed under chain of custody. The epidemiological characteristics of this cohort have been previously described in Part I of this study (*4*). From this total, N = 39 (32%) cases were selected based on a SAC_ED_ = 0.21 g/L. Figure 1 illustrates the stratification of SAC_ED_ and intra-ED TAT relative to sample selection. Regarding the estimation of PDFs, Table 1 summarizes the fitting results.

**Table 1:** Best fit of probability density functions

**Variable**	**Lognormal** **(SSQ)**	**Weibull** **(SSQ)**	**Gamma** **(SSQ)**
SAC_ED_	0.168050	0.036837	0.073868
TAT_intra_	159.0934	157.5233	143.5628
TAT_extra_	20.64071	9.151448	8.955764
SSQ – sum of squared differences. ED – emergency department. SAC_ED_ – serum alcohol concentrations at admission. TAT_intra_ – intra-ED turnaround time. TAT_extra_ – extra-ED turnaround time.			

Analysis of the deciles for the estimated SAC_ED_ PDF (with θ = 0.21 g/L) revealed a sub-optimal fit in the left tail of the distribution - the region most significantly associated with the generation of FN_pk_. Consequently, a compromise was applied by setting θ = 0.15 g/L. This corresponds to approximately 0.13 g/L after adjusting for SAC bias - a value where the elimination constant is roughly 80% of the ADH saturation point - resulting in an SSQ = 0.028957 (*8*). Final parameter details are provided in Table 2.

**Table 2:** Parameters of fitted probability density functions

**Variable**	**Distribution**	**location**	**scale**	**shape**
SAC_ED_	Weibull	0.15	1.431	1.808
TAT_intra_	Gamma	65.4	52.912	1.319
TAT_extra_	Gamma	30	13.766	1.591
ED – emergency department. SAC_ED_ - serum alcohol concentrations at admission. TAT_intra_ - intra-ED turnaround time. TAT_extra_ - extra-ED turnaround time.				

**Figure 2:**
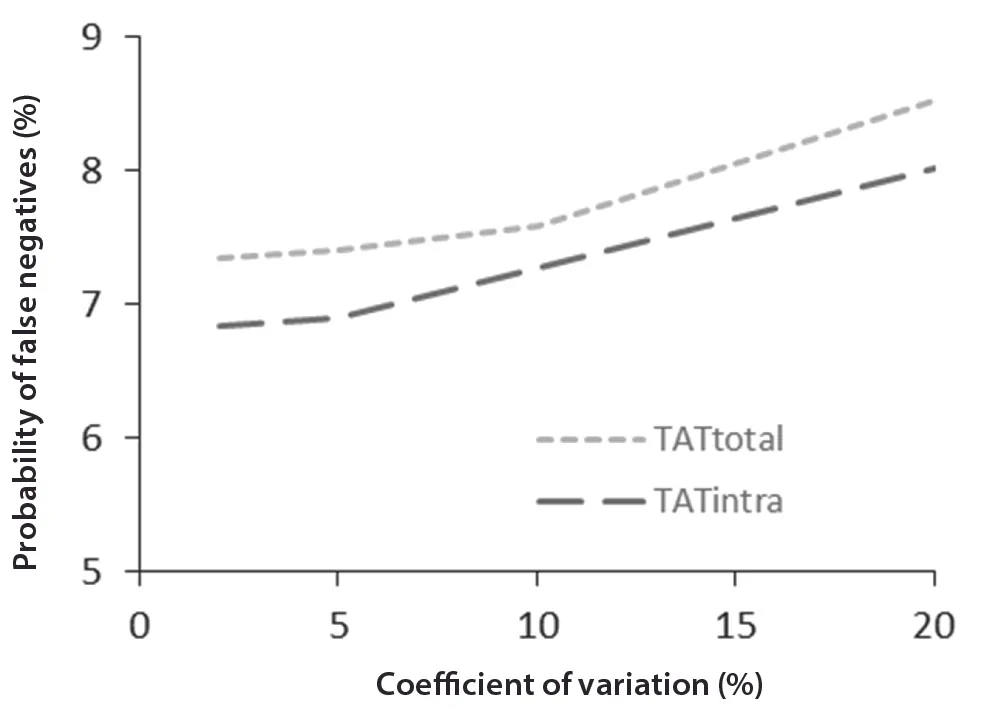
Effect of assay imprecision, measured as the coefficient of variation (CV), on the probability of pharmacokinetic false negatives (P(FN_pk_)). P(FN_pk_) was calculated based on either the intra-emergency department turnaround time (TAT_intra_) alone or the combined intra- and extra-ED TAT (TAT_total_).

Based on the parameters in Table 2, Monte Carlo simulations indicate a P(FN_pk_) = 7.0% when considering TAT_intra_ alone, and P(FN_pk_) = 7.6% when including TAT_extra_. Figure 2 shows the impact of CV values ranging from 2% to 25% on P(FN_pk_).

**Figure 3:**
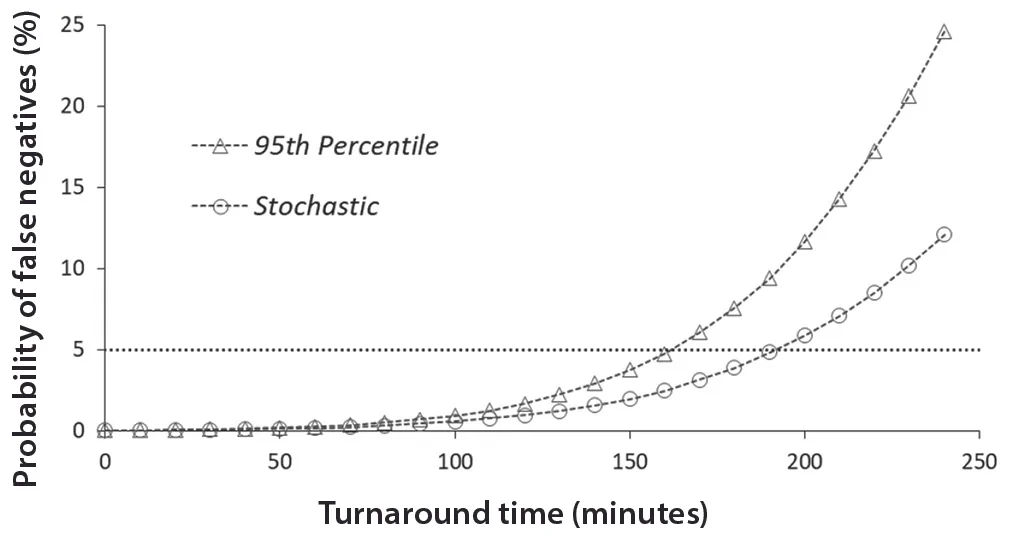
Relationship between the probability of false negatives (P(FN_pk_)) and the intra-Emergency Department turnaround time, calculated against a legal limit of alcohol intoxication of 0.5 g/L and an assay imprecision of 5% (TAT_L(CV)_). The graphic shows how short the TAT_L(CV)_ needs to be to ensure a given P(FN_pk_). P(FN_pk_) was calculated based on either the stochastic value (open circles) or the 95th percentile (open triangles) of the distribution of the elimination rate (β) of ethanol. The horizontal dotted line represents P(FN_pk_) = 5%.

The quality goals obtained assuming a legal positivity limit of 0.541 g/L (after adjustment for a CV of 5% uncertainty) are shown in Table 3. Figure 3 depicts the correlation between TAT_intra_ and P(FN_pk_) for a subject whose SAC_road_ is unknown at the time of ED admission.

**Table 3:** Quality goals for the intra-emergency department turnaround time calculated for subjects with rapid elimination kinetic (β*_95th_*) or random population value (β*_stochastic_*)

**Quality goal**	**Level of protection (%) against FN_pk_**	**Minimum serum alcohol at the scene (SAC_road_, g/L) protected from FN_pk_**	**TAT_intra_ (minutes)**	
**β*_95th_***	**β*_stochastic_***
	50	1.61	298	375
Minimum (TAT_60%_)	60	1.53	273	342
	70	1.45	245	306
Acceptable (TAT_80%_)	80	1.35	214	266
	90	1.22	169	213
Optimal (TAT_95%_)	95	1.12	134	171
	99	0.91	65	89
FNpk -pharmacokinetic false negative, *i.e.* a subject which is negativized by the prolonged waiting for the blood sampling in the emergency department. TATintra - intra-ED turnaround time. A legal limit of intoxication of 0.5 g/L adjusted for analytical imprecision of CV = 5% (final value 0.541 g/L) was applied.				

**Figure 4:**
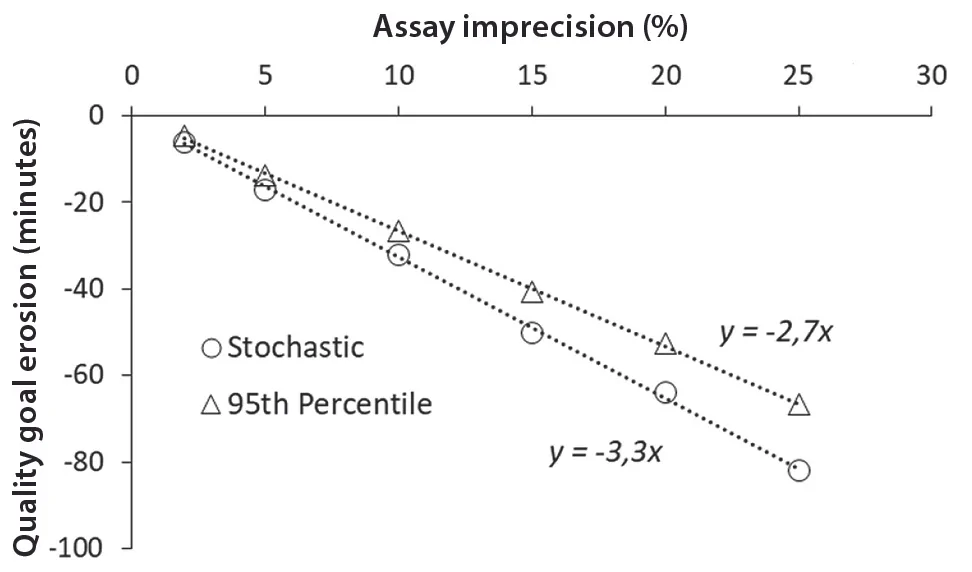
Effect of assay imprecision, measured as the coefficient of variation (CV), on the temporal erosion of the turnaround time (TAT_intra_) quality goal, calculated against a legal limit of alcohol intoxication of 0.5 g/L and an assay imprecision of 5%. Temporal erosion (measured in minutes) was calculated based on either the stochastic value (open circles; μ_stochastic_ ≈ 0.15 g/L/h) or the 95th percentile (open triangles; β_95th_ ≈ 0.2 g/L/h) of the ethanol elimination rate (β) distribution. Linear regression analysis reveals that for any TAT goal, there is a temporal erosion ranging from - 2.7 to - 3.3 minutes for every one-percentage-point increase in assay imprecision, depending on whether β is used to simulate a random individual (the stochastic value) or a rapid metabolizer (the 95th percentile of β).

Regression analysis indicates that for every one-percent increase in assay CV, the TAT quality goal is reduced by approximately 2.7 to 3.3 minutes. This variability depends on whether the calculation is based on the 95th percentile of the ethanol elimination rate (β_95th_ ≈ 0.2 g/L/h, a fixed value representing rapid metabolizers) or the stochastic population elimination (representing the random variation around the population mean μ_stochastic_ ≈ 0.15 g/L/h). This relationship is illustrated in Figure 4.

**Figure 5:**
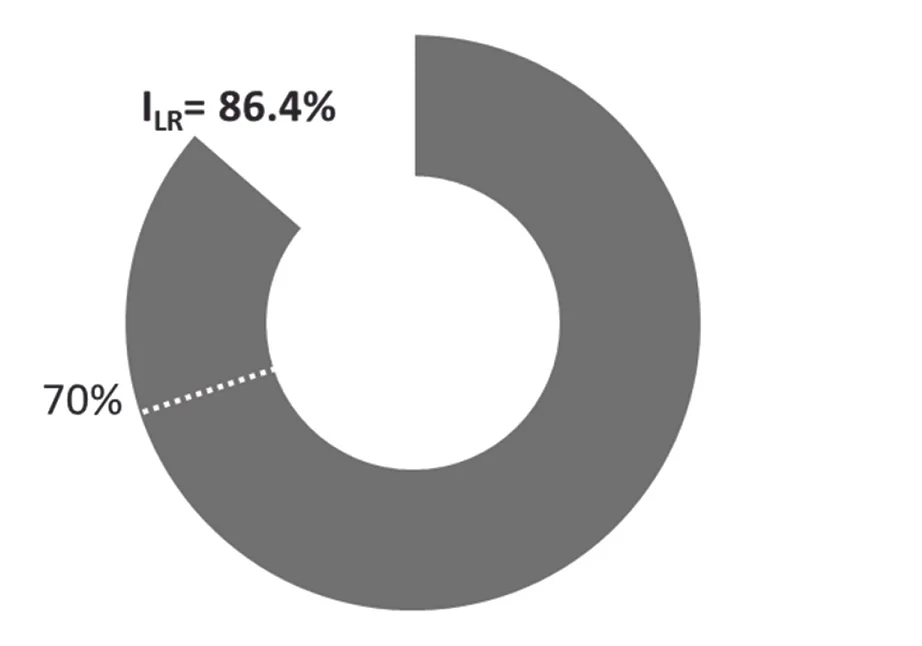
Graphical representation of the Index of Logistic Reliability (I_LR_). The full circle represents the average time elapsed from the car accident—the moment the subject was putatively positive - to the point of negativization relative to the legal limit of alcohol intoxication (LLI) of 0.5 g/L. The dark-gray shaded area indicates the time window available to perform blood sampling in the emergency department (ED) following the subject’s arrival at the hospital. The dashed white line represents the acceptability threshold of I_LR_ = 70%.

Regarding the efficiency of the extra-ED TAT phase, the I_LR_ = 86.4% (Figure 5). Therefore, based on a minimum target I_LR_ of 70%, the P_LS_ = 96.6%,

## Discussion

The Monte Carlo simulation provides a robust framework for defining objective quality goals of timelines in forensic testing for drink-driving. Numerical simulations become indispensable when experimental evidence is neither ethical nor practically feasible. This makes them an essential tool in the investigation of extra-analytical quality, where outcome studies - while highly desirable for producing high-value information to define quality goals - remain extremely difficult to implement (*9*, *10*).

By modeling the stochastic nature of ethanol pharmacokinetics and the analytical uncertainty of the laboratory assay, we have translated TAT into measures of forensic reliability. Therefore, TAT is not merely a KPI of efficiency but a critical determinant of legal admissibility. This completes the characterization of the reliability paradigm for forensic procedures in medical emergency settings, alongside preanalytical procedures and assay robustness (*11*, *12*).

The simulation analysis of epidemiological findings from Part I revealed that P(FN_pk_) was as much as 7.0% when considering the intra-ED phase alone and increased marginally to 7.6% when the extra-ED phase was included. This suggests that approximately one in thirteen drivers, despite being above the legal limit at the time of the incident, may test negative at the time of sampling due to metabolic decay. We may consider this result satisfactory if we assume an arbitrary goal of 5% (recalling Eq. 8), although there is no evidence in the literature establishing such a value as an objective quality goal.

Thus, to drive process improvement through truly objective criteria, we defined three tiers of quality goals for the TAT_intra_ based on SAC_road_ data from a population of drivers whose blood alcohol content was known through roadside controls. In this manner, ED performance is calibrated against the specific composition of the individuals potentially prompting a CoC. For the evaluation of ED performance, we compared these goals with the 95th percentile calculated for the TAT_intra_. While this percentile is certainly more representative of execution performance, it is also more susceptible to the influence of extreme values, given that the TAT PDF is strongly right-skewed. Consequently, further investigation is warranted to assess the stability of different percentiles relative to their ability to represent process performance, particularly regarding P(FN_pk_).

Notwithstanding, our current 95th percentile of 294 minutes observed in Part I (95% CI: 271–320 minutes) rests entirely between the TAT_80%_ and the TAT_60%_. We can therefore state that our performance falls within an acceptability range that guarantees coverage of a SAC_road_ of at least 1.53 g/L, which is slightly above the median of 1.48 g/L observed in Part I. This figure appears as acceptable as a SAC of 1.53 g/L in the ED was certainly higher at the time of the incident, that is SAC_road_. However, it must be recalled that in our case series, 7.4% of subjects had a SAC between 0.1 and 0.5 g/L and 64.5% had values below 0.1 g/L. Therefore, bearing in mind that the logic of this “protection” is to ensure that operating within such a maximum TAT_intra_ (*i.e.,* the 95th percentile) guarantees an 80% to 60% probability that a random subject remains above the legal limit at the time of sampling, it is evident that a significant portion of the observed negatives are likely false negatives. It must be also considered that under the most stringent quality goal parameters, our performance significantly exceeds the minimum acceptable TAT_60%_ of 273 minutes. Therefore, process improvement is clearly necessary.

To develop an improvement plan, the TAT_intra_ stratification from Part I (Table 1) indicates that the most actionable phase is the transmission of the request for CoC testing. This phase alone can account for up to nearly 150 minutes, which is likely to be merely a waiting time for almost 50% of CoC of drink-driving cases that are represented by lower-severity triage codes. Importantly, establishing these quality goals does not imply that forensic sampling should ever compete with active medical resuscitation. Patient care and stabilization remain the absolute ethical and clinical priorities in the ED. Instead, these performance benchmarks target systemic, administrative bottlenecks - such as the request-transmission phase - which often leave stable, lower-severity patients waiting unnecessarily, thereby inadvertently degrading vital forensic evidence.

Regarding the extra-ED phase, another part of potential improvement, our benchmark indices show that with an ILR of 86.4%, patient transport consumes less than 20% of the “metabolic time” to reach L. Thus, the ED has over 80% of the available time to perform the blood draw before the subject tests negative. If we set a minimum quality goal for transport at an I_LR_ of 70%, the P_LS_ of 96.6% indicates that the transport process is fully capable of meeting this requirement. These data align well with the 0.6 percentage point increase in P(FN_pk_) observed when adding TAT_extra_ to TAT_intra_, objectively excluding the logistical process from the primary improvement path.

A key advantage of numerical simulations is the ability to model diverse clinical and analytical scenarios, which enabled us to investigate the relationship between TAT and the assay CV. Our results demonstrate that higher CV values necessitate shorter sampling times to maintain an equivalent level of forensic certainty. Indeed, the CV acts as a “hidden delay” that erodes the quality goal by raising the ‘protected’ LLI or, equivalently, by blurring the measured SAC. Given an erosion rate of 2.7 to 3.3 minutes for every one-percent increase in CV, a 2% CV (typical of chromatographic methods) offers no substantial advantage over the 5% CV afforded by modern, fully automated ADH assays. In contrast, a 10% CV reduces the TAT quality goals by approximately 30 minutes across all tiers, while a 20% CV results in a nearly 60-minute reduction. Beyond the analytical uncertainty introduced by the assay CV, the model is similarly sensitive to individual metabolic variations, particularly in the “worst-case biological scenario” of rapid clearers simulated by the 95th percentile of the elimination rate (β_95th_). As shown in Table 3, when shifting from a stochastic population distribution (μ_stochastic_ ≈ 0.15 g/L/h) to a rapid clearance rate (β_95th_ ≈ 0.20 g/L/h), the allowable time window to capture evidence shrinks significantly from 171 to 134 minutes. This explicitly demonstrates how an increasing β compounds the “hidden delay” of analytical error and drastically escalates the P(FN_pk_). These findings are of particular relevance given that current regulations do not distinguish between the clinical and forensic applications of enzymatic ADH assays (*12*). Indeed, even though the enzymatic ADH testing is not acceptable for forensic confirmation due to its known limitations, nevertheless, establishing strict operational timelines remains vital to safeguard the reliability of the initial screening phase within the clinical laboratory framework before eventual chromatographic confirmation (*12*).

The critical need for workflow timeliness is further amplified by regional legal frameworks. In countries like Italy, retrograde extrapolation (back-calculation) is judicially inadmissible, meaning that a delayed sample cannot be legally reconstructed. Consequently, any screening result where SAC < 0.5 g/L is strictly dismissed as legally negative and is not sent for confirmation, unless a zero-tolerance policy applies. Conversely, in jurisdictions where retrograde extrapolation is permitted, achieving a rapid intra-ED TAT remains scientifically vital. Ensuring the blood draw occurs before the blood alcohol levels decay into non-linear elimination kinetics SAC < 0.21 g/L is a prerequisite for executing a mathematically valid and safe back-calculation.

Concerning the limitations of this study, as a simulation its power primarily depends on the goodness-of-fit of the PDFs to the observed data. Given the relatively small sample size, the accuracy of the PDF modeling may have been constrained, particularly within the left tail of the distribution where P(FN_pk_) cases originate. Consequently, the defined quality goals may lack perfect centering. To address this concern, it must be noted that P(FN_pk_) and TAT quality goals do not necessarily coincide. The reason is that these two measures approach the same issue from opposite perspectives. Specifically, P(FN_pk_) is a backward estimation based on “aftermath” data; at this stage, it is difficult to determine which samples falling below the LLI (especially if also below the limit of quantitation) were originally positive but turned negative due to waiting times. Conversely, quality goals are established *via* forward calibration on the actual population expected during roadside controls - the population most likely to generate CoC events. The same applies when considering the difference between P(FN_pk_) calculated on TAT_intra_ and TAT_total_, respect to the I_LR_. Therefore, the suggested strategy is to use P(FN_pk_) as a starting point to evaluate the forensic reliability of the ED and subsequently rely on calibrated quality goals once the population has been characterized for prediction.

Finally, a fundamental limitation of the present study concerns its single-center retrospective design and the specific dataset used to configure the simulation model. While the Monte Carlo framework incorporates a massive number of stochastic iterations to capture analytical and physiological variability, the underlying PDFs are fundamentally reflective of the specific operational workflows, logistical bottlenecks, and patient demographics of a single metropolitan university hospital. Consequently, the validity, predictive power, and target thresholds (TAT_95%_, TAT_80%_, TAT_60%_) of this quality-goal model must be rigorously evaluated on an independent set of samples. External validation using discrete data matrices collected from diverse emergency frameworks and geographical jurisdictions is essential. Testing the model against such independent datasets will confirm whether these algorithmic quality definitions can be safely generalized as standard performance indicators across the broader clinical and forensic toxicology community.

In conclusion, this two-part study provides a quantitative framework for assessing forensic equity in the ED. Based on numerical simulations of collected epidemiological data, this offers an unprecedented approach in laboratory medicine literature that merges medico-legal reliability with patient safety. Future research should expand upon this model, ideally through prospective multi-center studies integrating extensive data from both roadside controls and diverse hospital settings.

## Data Availability

The data generated and analyzed in the presented study are available from the corresponding author on request.
